# Concordance and Reproducibility of Melanoma Staging According to the 7th vs 8th Edition of the *AJCC Cancer Staging Manual*

**DOI:** 10.1001/jamanetworkopen.2018.0083

**Published:** 2018-05-18

**Authors:** Joann G. Elmore, David E. Elder, Raymond L. Barnhill, Stevan R. Knezevich, Gary M. Longton, Linda J. Titus, Martin A. Weinstock, Margaret S. Pepe, Heidi D. Nelson, Lisa M. Reisch, Andrea C. Radick, Michael W. Piepkorn

**Affiliations:** 1 Department of Medicine, David Geffen School of Medicine, University of California, Los Angeles; 2 Department of Pathology and Laboratory Medicine, Hospital of the University of Pennsylvania, Philadelphia; 3 Department of Pathology, Institut Curie, Paris, France; 4 Paris Sciences and Lettres Research University, Paris, France; 5 Faculty of Medicine, University of Paris Descartes, Paris, France; 6 Pathology Associates, Clovis, California; 7 Program in Biostatistics and Biomathematics, Fred Hutchinson Cancer Research Center, Seattle, Washington; 8 Department of Epidemiology, Geisel School of Medicine at Dartmouth, Hanover, New Hampshire; 9 Department of Pediatrics, Geisel School of Medicine at Dartmouth, Hanover, New Hampshire; 10 Norris Cotton Cancer Center, Lebanon, New Hampshire; 11 Center for Dermatoepidemiology, Providence Veterans Affair Medical Center, Providence, Rhode Island; 12 Department of Dermatology, Brown University, Providence, Rhode Island; 13 Department of Epidemiology, Brown University, Providence, Rhode Island; 14 Department of Medical Informatics and Clinical Epidemiology, School of Medicine, Oregon Health and Science University, Portland; 15 Department of Medicine, School of Medicine, Oregon Health and Science University, Portland; 16 Department of Medicine, University of Washington School of Medicine, Seattle; 17 Dermatopathology Northwest, Bellevue, Washington

## Abstract

**Importance:**

The recently updated American Joint Committee on Cancer (AJCC) classification of cancer staging, the *AJCC Cancer Staging Manual*, 8th edition (*AJCC 8*), includes revisions to definitions of T1a vs T1b or greater. The Melanoma Pathology Study database affords a comparison of pathologists’ concordance and reproducibility in the microstaging of melanoma according to both the existing 7th edition *(AJCC 7*) and the new *AJCC 8*.

**Objective:**

To compare *AJCC 7* and *AJCC 8* to examine whether changes to the definitions of T1a and T1b or greater are associated with changes in concordance and reproducibility.

**Design, Setting, and Participants:**

In this diagnostic study conducted as part of the national Melanoma Pathology Study across US states, 187 pathologists interpreting melanocytic skin lesions in practice completed 4342 independent case interpretations of 116 invasive melanoma cases. A consensus reference diagnosis and participating pathologists’ interpretations were classified into the Melanocytic Pathology Assessment Tool and Hierarchy for Diagnosis class IV (T1a) or class V (≥T1b) using both the *AJCC 7 *and *AJCC 8* criteria.

**Main Outcomes and Measures:**

Concordance with consensus reference diagnosis, interobserver reproducibility, and intraobserver reproducibility.

**Results:**

For T1a diagnoses, participating pathologists’ concordance with the consensus reference diagnosis increased from 44% (95% CI, 41%-48%) to 54% (95% CI, 51%-57%) using *AJCC 7* and *AJCC 8* criteria, respectively. The concordance for cases of T1b or greater increased from 72% (95% CI, 69%-75%) to 78% (95% CI, 75%-80%). Intraobserver reproducibility of diagnoses also improved, increasing from 59% (95% CI, 56%-63%) to 64% (95% CI, 62%-67%) for T1a invasive melanoma, and from 74% (95% CI, 71%-76%) to 77% (95% CI, 74%-79%) for T1b or greater invasive melanoma cases.

**Conclusions and Relevance:**

Melanoma staging in *AJCC 8* shows greater reproducibility and higher concordance with a reference standard. Improved classification of invasive melanoma can be expected after implementation of *AJCC 8*, suggesting a positive impact on patients. However, despite improvement, concordance and reproducibility remain low.

## Key Points

**Question:**

Do changes to the American Joint Committee on Cancer (AJCC) cancer staging system for melanoma improve concordance and reproducibility for invasive melanomas?

**Findings:**

In this diagnostic study, melanoma staging in the *AJCC Cancer Staging Manual*, 8th edition, showed greater reproducibility and higher concordance with a reference standard than melanoma staging in the *AJCC Cancer Staging Manual*, 7th edition.

**Meaning:**

Improved classification of invasive melanoma can be expected after implementation of the *AJCC Cancer Staging Manual*, 8th edition, suggesting a positive impact on patients.

## Teaser

This diagnostic accuracy study evaluates whether changes to the definitions of T1a and T1b or greater invasive melanoma staging between the *AJCC Cancer Staging Manual* 7th and 8th editions by the American Joint Committee on Cancer (AJCC) led to changes in the concordance and reproducibility of pathologists’ readings of melanocytic skin biopsies compared with a consensus reference standard.

## Introduction

Disease subclassification according to the *AJCC Cancer Staging Manual* by the American Joint Committee on Cancer (AJCC) is the customary and prevalent mode for stratifying patients with melanoma to estimate prognosis, determine appropriate surgical intervention, and assess eligibility for adjuvant therapies and clinical trials. The process presupposes that pathologists’ application of the AJCC histopathological criteria to individual cases of melanoma is accurate and reproducible.

However, in the field of melanoma, there are only limited analyses quantifying the degree of reproducibility of AJCC microstaging between pathology observers.^1^ Extensive variability has been noted among pathologists in the diagnosis of invasive melanoma.^2,3,4,5,6,7^ One of the largest studies,^2^ our previously published Melanoma Pathology Study (M-Path) of 187 US pathologists, found less than 50% agreement between pathologists and a consensus-derived reference diagnosis of T1a invasive melanoma, with improvement to 72% concordance for invasive melanoma T1b or greater. Similarly, M-Path findings revealed only 46% interobserver agreement for T1a invasive melanoma, and 77% agreement for T1b or greater melanomas.^2^

The previous study^2^ was conceived and executed in the context of the *AJCC Cancer Staging Manual*, 7th edition (*AJCC 7*) staging system. Across interpretations at 2 points, pathologists’ intraobserver reproducibility reached 63% for T1a melanomas and 83% for T1b or greater melanomas. Given the updated classification in the *AJCC Cancer Staging Manual*, 8th edition (*AJCC 8*), particularly with changes in definitions of T1a vs T1b or greater, the M-Path database enables a new comparison of pathologist concordance with a reference standard and reproducibility in the microstaging of melanoma according to both the existing *AJCC 7 *and the current *AJCC 8*.^8,9^ Briefly, in *AJCC 8*, the depth for stage T1a is established at 0.8 mm, rather than 1.0 mm, and the presence of ulceration continues to contribute to stage modification, but mitoses do not. In addition, the reporting of Breslow thickness is limited to intervals of tenths of a millimeter rather than hundredths. We assess whether changes in criteria in the newer *AJCC 8 *are associated with changes in concordance and reliability, and whether observer interpretations of histological alterations within melanocytic lesions are reliable in the context of the demands of microstaging and its consequences per the AJCC schema.

## Methods

### Study Design

The data used in this diagnostic study are derived from the M-Path study,^2^ which was described previously. Practicing pathologists from 10 US states who actively interpreted melanocytic skin biopsy lesions as part of their usual clinical practice and planned to continue practicing for a minimum of 2 subsequent years were invited to participate. This study was approved by the institutional review boards of Dartmouth College, the Fred Hutchinson Cancer Research Center, Oregon Health and Science University, and the University of Washington. Informed consent was obtained from every participating pathologist using an online platform.

Each pathologist was randomized to interpret the same set of melanocytic skin biopsy cases on 2 occasions, at least 8 months apart. The study cases (n = 240) were assembled into 5 sets of 48 cases, each represented by a single glass slide. Each set included the full spectrum of melanocytic skin lesions (eg, from benign to invasive melanoma).

Participating pathologists independently reviewed the same cases using the same glass slides. Participants entered diagnostic interpretations into an online Melanocytic Pathology Assessment Tool and Hierarchy for Diagnosis (MPATH-Dx) histology form for each case, choosing from a diverse and comprehensive list of more than 50 diagnostic terms. We asked participants to assume that the single glass slide for each case was representative of the entire lesion and that the margin was involved (irrespective of whether it involved the biopsy margin). Research analysts subsequently mapped diagnostic interpretations into 1 of 5 diagnostic classes according to the MPATH-Dx mapping scheme.^10^ Examples of diagnostic terms for each class and suggested treatment recommendations, provided under the assumption that specimen margins are positive, are depicted in Table 1. Because the *AJCC 8* criteria changes only affect MPATH-Dx classes IV (T1a) and V (≥T1b), this article focuses on the distinction between invasive melanomas exclusively.

**Table 1.** The MPATH-Dx Reporting Schema for Melanocytic Skin Lesion Classification Into 5 Diagnostic Classes, as Used in This Study^a^

MPATH-Dx Class	Perceived Risk for Progression	Suggested Intervention^b^	Examples
0	Incomplete study due to sampling or technical limitations	Repeat biopsy or short-term follow-up	NA
I	Very low risk	No further treatment	Common melanocytic nevus; blue nevus; mildly dysplastic nevus
II	Low risk	Narrow but complete excision (<5 mm)	Moderately dysplastic nevus; Spitz nevus
III	Slightly higher risk, greater need for intervention	Complete excision with ≥5-mm but <1-cm margins	Severely dysplastic nevus; melanoma in situ; atypical Spitz tumor
IV	Substantial risk for local or regional progression	Wide local excision with ≥1-cm margins	Thin invasive melanomas (eg, T1a)
V	Greatest risk for regional and/or distant metastases	Wide local excision with ≥1-cm margins; consideration of staging sentinel lymph node biopsy; adjuvant therapy	Thicker invasive melanoma (eg, T1b, stage ≥2)

Abbreviations: MPATH-Dx, Melanocytic Pathology Assessment Tool and Hierarchy for Diagnosis; NA, not applicable.

^a^ Adapted from Piepkorn et al.^10^ These examples of suggested interventions were developed at the beginning of the study, are presented for consideration only, and may be out of date or controversial in some instances. Additional consensus development should proceed before these guidelines are adopted for general use, and they should be adapted according to individual national circumstances. In particular, the suggestions for melanoma should follow published national guidelines as most recently updated.

^b^ Assuming representative sampling of the lesion.

Before data collection, a panel of 3 experienced dermatopathologists independently reviewed the hematoxylin-eosin–stained glass slides for each case followed by consensus review using a modified Delphi approach.^11,12^ This process was used to develop a consensus diagnosis for each of the M-Path study cases. Only 116 cases of invasive melanoma, as defined by the consensus diagnosis, were considered in this analysis. Three cases included in the original M-Path study as class IV were excluded here because classification was based on a treatment recommendation of wide excision but these cases were assessed as melanocytic lesions of uncertain malignant potential.

### Statistical Analysis

For each case, the consensus reference diagnosis and the participating pathologists’ interpretations were classified into the MPATH-Dx class IV (T1a) or class V (≥T1b) using both the *AJCC 7* and *AJCC 8* criteria.^8,9^ Accuracy outcome measures included overinterpretation, underinterpretation, and concordance of participant interpretations with the relevant (*AJCC 7* or *AJCC 8*) reference diagnosis. We defined overinterpretation as diagnosing cases at a higher diagnostic class than the reference diagnosis, and underinterpretation as diagnosing cases at a lower diagnostic class than the reference diagnosis. Interpretations in agreement with the reference diagnosis were concordant. Confidence intervals accounted for both within-participant and across-participant variability by using variance estimates of the following form:

{var(ratep) + [ave(ratep) × (1−ave(ratep))]/nc}/np,

where ave(ratep) is the average rate among pathologists, var(ratep) is the sample variance of rates among pathologists, nc is the number of cases interpreted by each pathologist, and np is the number of pathologists. Logistic regression models were used to test for a difference in accuracy between *AJCC 7–* and *AJCC 8–*based mappings. Models used robust estimators of the variance to account for correlation of case interpretations from the same pathologist.

The reproducibility of participating pathologists’ interpretations were assessed as both intraobserver and interobserver concordance. Interobserver concordance considered all pairs of interpretations of the same invasive disease case by 2 different pathologists, and the proportion of those pairs for which interpretations were in the same diagnostic class was calculated. Although cases were restricted to those with invasive melanoma by consensus reference diagnosis, participating pathologist interpretations could include diagnoses in other noninvasive MPATH-Dx classes. Confidence intervals for interobserver concordance rates were bootstrap percentile intervals, and tests for differences between *AJCC 7–* and *AJCC 8–*based mappings used a Wald statistic based on the bootstrap standard error of the difference. A total of 3000 bootstrap samples were obtained by participant-level sampling with replacement and generation of all possible pairs of distinct sample participants for each sample.

For intraobserver concordance among the 118 participants who interpreted the same glass slides on 2 occasions, we calculated the proportion of cases with both interpretations in the same diagnostic class. Confidence intervals for intraobserver concordance rates used a logit transformation and robust standard error that accounted for clustering at pathologist level. Logistic regression models were used to test for a difference in intraobserver concordance between *AJCC 7–* and *AJCC 8–*based mappings. All *P* values correspond to 2-tailed tests and differences with *P* < .05 were considered to be statistically significant. Analyses were performed using Stata statistical software (StataCorp), version 14.

## Results

The 116 skin biopsy cases defined as invasive melanoma per the consensus reference diagnosis included 55 cases (47%) of T1a invasive melanoma and 61 cases (53%) of T1b or greater using *AJCC 7*. When *AJCC 8* staging criteria were applied, the consensus reference diagnosis was upgraded from T1a to T1b or greater for 4 of 55 cases (7%) and downgraded from T1b or greater to T1a for 19 of 61 cases (31%). The reclassification of invasive cases by consensus reference diagnosis under *AJCC 8* resulted in 70 T1a cases (60%) and 46 cases (40%) of T1b or greater.

Of 301 eligible pathologists, 187 (62%) enrolled and completed independent interpretations. In the first round of interpretations, the pathologists completed 4342 independent case interpretations of the invasive melanoma cases. Similar to the aforementioned movement in consensus reference diagnoses, participant diagnoses were upgraded from T1a to T1b or greater for 136 of 1229 T1a assessments (11%) and downgraded from T1b or greater to T1a for 467 of 1841 assessments (25%).

As shown in Table 2, concordance and reproducibility were improved when using the *AJCC 8* criteria vs the earlier *AJCC 7* criteria. With regard to T1a diagnoses, participating pathologists’ concordance with the consensus reference diagnosis increased from 44% (95% CI, 41%-48%), using *AJCC 7* criteria, to 54% (95% CI, 51%-57%), using *AJCC 8* criteria. The concordance for T1b or greater cases increased from 72% (95% CI, 69%-75%) to 78% (95% CI, 75%-80%). The increased concordance associated with using the *AJCC 8* reduced both underinterpretation and overinterpretation.

**Table 2.** Changes in Concordance, Interobserver Agreement, and Intraobserver Reproducibility When Comparing *AJCC 7* With *AJCC 8*

*AJCC Cancer Staging Manual* Edition	Total Invasive Melanoma Cases for Consensus, No.	% (95% CI)							
		Concordance With Consensus Reference Diagnosis				Interobserver Agreement		Intraobserver Reproducibility for Same Case at 2 Time Points	
		Underinterpretation	Concordance	*P* Value^a^	Overinterpretation	Concordance	*P* Value^a^	Reproducibility	*P* Value^a^
*AJCC 7*									
T1a (MPATH-Dx class IV)	55	46 (43-50)	44 (41-48)		9 (8-12)	41 (39-44)		59 (56-63)	
T1b or greater (MPATH-Dx class V)	61	28 (25-31)	72 (69-75)		NA	67 (64-69)		74 (71-76)	
*AJCC 8*									
T1a	70	39 (36-42)	54 (51-57)	<.001	7 (6-8)	51 (48-53)	<.001	64 (62-67)	.006
T1b or greater	46	22 (20-25)	78 (75-80)	<.001	NA	69 (66-73)	.02	77 (74-79)	.11

Abbreviations: *AJCC 7*, *AJCC Cancer Staging Manual*, 7th edition; *AJCC 8*, *AJCC Cancer Staging Manual*, 8th edition; MPATH-Dx, Melanocytic Pathology Assessment Tool and Hierarchy for Diagnosis; NA, not applicable.

^a^ *P* values for test of concordance, interobserver agreement, and intraobserver reproducibility rate differences between *AJCC 7–* and *AJCC 8–*based mappings.

The intraobserver reproducibility of diagnoses also improved when using the *AJCC 8* criteria, increasing from 59% (95% CI, 56%-63%) to 64% (95% CI, 62%-67%) for T1a invasive melanoma, and from 74% (95% CI, 71%-76%) to 77% (95% CI, 74%-79%) for T1b or greater invasive melanoma cases. Average pairwise-interobserver agreement increased from 41% (95% CI, 39%-44%) to 51% (95% CI, 48%-53%) for T1a cases, and from 67% (95% CI, 64%-69%) to 69% (95% CI, 66%-73%) for T1b or greater cases.

## Discussion

This analysis provides data that the new *AJCC 8* criteria may lead to improved concordance and reproducibility among pathologists in the classification of invasive melanoma, although the size of this effect is modest. One explanation of the improvement in concordance of pathological staging of T1a and T1b melanoma in *AJCC 8* is the change in stage T1 subgroups and criteria from *AJCC 7*. In *AJCC 7*, the criteria for T1b were presence of dermal mitotic activity, Breslow thickness, or epidermal ulceration,^8^ whereas in *AJCC 8*, the primary determinants for T1a vs T1b were Breslow thickness and ulceration, with the elimination of mitotic activity.^9^

In *AJCC 8*, T1b is now defined by Breslow thickness 0.8 mm or greater or ulceration in melanomas smaller than 0.8 mm. Because recognition of mitoses in thin melanomas is considered potentially unreliable^13^ and the recording of Breslow thickness more reliable,^14^ one would expect to find greater reliability of both T1a and T1b classification in the *AJCC 8* staging. In fact, our results correspond exactly to this presupposed increase in reliability of classification of T1a and T1b in *AJCC 8*. A retrospective restaging of the Netherlands Cancer Registry database also reported a modest improvement in stratification of pT1 melanoma associated with the implementation of *AJCC 8* criteria.^15^

### Limitations

Limitations of the study include interpretation of a single slide (although participants were asked to assume the slide was representative), use of a testing environment rather than a practice setting, and inability to obtain second opinions and clinical histories. Also, there is no established method to define a gold-standard diagnosis; therefore, improvement in concordance with an expert-defined reference should not necessarily be interpreted as improvement in accuracy. We chose to use the consensus of 3 experienced pathologists because this approach could be replicated in clinical practice. Finally, the relative proportions of cases used for this study are not representative of the population.^16^ Strengths include a large number of participating pathologists reviewing the same glass slides on 2 occasions and the ability to assess both concordance with a reference and reproducibility.

## Conclusions

Our results suggest that the changes in the AJCC staging will likely have a positive effect on patients. The consequences of melanoma staging to patients are substantial. Among these are patients’ perceptions of long-term implications to their health as determined by the particular stage assigned at diagnosis, economic consequences of health care services, and the magnitude of surgical interventions indicated by the staging classification (eg, size of wide local resection, eligibility for sentinel lymphatic mapping, and implications for other therapies). In view of these clinical ramifications, even modest improvements of 6% to 10% in diagnostic concordance resulting from changes from *AJCC 7* to *AJCC 8* are important. However, despite improvement, concordance and reproducibility remain low and suggest that conventional histopathology has been parsed to a degree that falls below the limits of reliability for the demands and consequences of the staging schemata that have evolved over time.
